# Cats as sentinels of mammal exposure to H5Nx avian influenza viruses: a seroprevalence study, France, December 2023 to January 2025

**DOI:** 10.2807/1560-7917.ES.2025.30.12.2500189

**Published:** 2025-03-27

**Authors:** Pierre Bessière, Jessie Brun, Brandon Hayes, Amélie Marchand, Laura Lebouteiller, Sébastien-Mathieu Soubies, Marie-Christine Cadiergues, Jean-Luc Guérin

**Affiliations:** 1 IHAP, Université de Toulouse, INRAE, ENVT, Toulouse, France; 2 Small Animal Clinic, Université de Toulouse, ENVT, Toulouse, France

**Keywords:** avian influenza, spillover, zoonoses, cats, H5N1, infection, pets

## Abstract

Circulation of clade 2.3.4.4b highly pathogenic avian influenza H5Nx viruses has intensified in recent years, increasing epizootics and mammalian exposure. Cats, bridging wild and domestic environments, are key for studying cross-species transmission. To assess their exposure in France, we screened 728 outdoor cats (December 2023–January 2025). Seropositivity was 2.6% (19/728), with an estimated seroprevalence at 1.8%. Absence of hunting behaviour was a significant protective factor. These findings highlight high recent exposure and the need for targeted surveillance in cats.

Highly pathogenicity avian influenza viruses (HPAIV) H5Nx of clade 2.3.4.4b have circulated extensively in recent years and become enzootic in many regions since 2020 [1]. This increased circulation has led to greater exposure of mammals to these viruses [2]. Cats are known to be susceptible to infection by HPAIV H5Nx, with cases — sometimes serious or fatal — reported following contact with infected birds and the ingestion of contaminated raw meat or milk [2,3]. A Dutch study recently identified a 11.8% and 0.46% seropositivity rate among stray and domestic cats respectively, highlighting their potential role as sentinels for carnivorous mammals’ exposure to HPAIV H5Nx [4].

Our study aimed to provide an estimation of the seroprevalence against two HPAIV H5Nx types in France. 

## Sample collection and serological analysis

Over a 14-month period from December 2023 to January 2025, serum samples were collected in 44 veterinary practices from 728 cats (642 domestic and 86 stray) living in mainland France (Figure). Each sample was accompanied by a questionnaire documenting the cat’s lifestyle. De-identified data were securely transferred and stored. All cats had outdoor access and were located outside France’s major metropolitan areas (to include only animals with potential exposure to domestic avifauna), with an average age of 6.6 years. There were 329 female and 371 male and sex was unknown for 28 animals. Sixty-seven per cent of the cats (488/728) lived in départements that the French Ministry of Agriculture has classified as at-risk based on the presence of wild bird migration corridors, wetlands where birds like to rest, and/or a very high density of poultry farms [5]. We initially screened the sera using a commercial anti-H5 ELISA kit (ID Screen Influenza H5 Antibody Competition 3.0 Multi-species, Innovative diagnostic, Grabels, France). Competition percentage (CP) was calculated as: CP = 100 × (OD_sample_/OD_negative control_). Samples with CP ≤ 50% were considered positive; samples with CP ≥ 60% were considered negative; samples with CP between between 50% and 60% were considered inconclusive. Positive or inconclusive samples were further analysed by virus-serum neutralisation assay, using two viral strains: an HPAIV H5N1 of clade 2.3.4.4b (A/Mule duck/France/21348/2021) and a low pathogenic avian influenza virus (LPAIV) H5N3 (A/Duck/Italy/775/2004).

**Figure:**
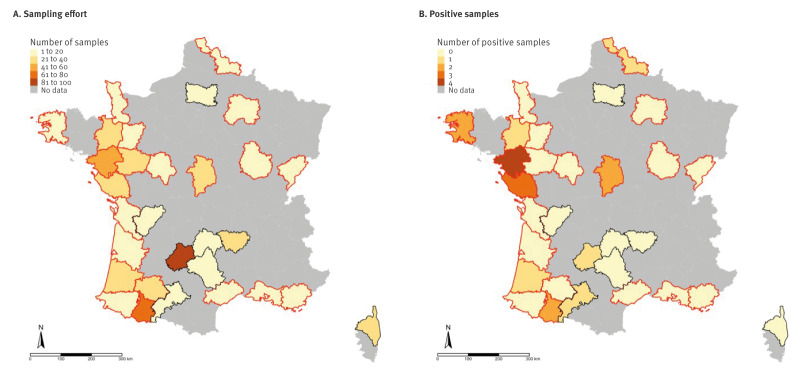
Geographical distribution of cats tested for H5Nx avian influenza viruses, France, December 2023–January 2025 (n = 728)

Départements defined as at-risk are indicated by a red border.

All ELISA-positive or inconclusive sera were virus-serum neutralisation-positive. Overall seropositivity was 2.6% (19/728), with titres ranging from 1:40 to 1:1,280. Only two sera had a higher titre against LPAIV H5N3 than against HPAIV H5N1. Thus, the majority of cats were exposed to clade 2.3.4.4b H5N1 viruses, although exposure to LPAIV strains endemic in France could not be ruled out. Seropositivity in départements classified as at-risk was slightly higher at 3.3%, although no positive samples were identified in 15 of the 23 at-risk départements. Seropositive cats were detected in two of the seven not at-risk département (Figure). Information on the cats (age, location, sampling date, time spent outdoor, hunting behaviour frequency as perceived by the owners, etc.) and test results (ELISA results and neutralisation assay antibody titres) is available in the Supplement. We estimated seroprevalence via Rogan–Gladen estimation using literature-informed test specificity and sensitivity (0.99 and 0.90, respectively) [6,7].

## Risk factor analysis

Estimated seroprevalence was 1.8% (95% confidence interval (CI): 0.66–3.4). Département at-risk status was not significantly associated with H5 seropositivity (p = 0.14) via Fisher’s exact test. An initially significant association was seen between hunting and H5 positivity (p = 0.043); however, this result failed to remain significant after adjustment for multiple comparisons (adjusted p = 0.13). When analysing only the 642 owned cats, we did not identify any significant association between hunting behaviour and H5 positivity (p = 0.13). Since stray status and hunting behaviour are inherently associated, with stray cats more frequently being hunters (reflecting the reality that hunting is their primary source of nourishment), we merged these categories into a single variable. Firth logistic regression indicated that owned, non-hunting cats had 94% lower odds of being H5-seropositive than stray cats (odds ratio (OR) = 0.06; 95% CI: 0–0.53; p = 0.01) (Table). Being from a département classified as at-risk showed a trend towards increased risk, but this did not reach statistical significance (p = 0.09), although there were more than three times as many at-risk départements as non-risk ones (23 versus seven). These findings suggest individual behaviours, particularly hunting, may drive H5 exposure more than geographical risk classification alone. Assessing additional predictors, such as habitat type (e.g. urban or rural), and the degree of bird exposure (e.g. owning backyard poultry), could further clarify the observed trends.

**Table:** Predictors of influenza A(H5) seropositivity in cats, France, December 2023–January 2025 (n = 728)

Variable	OR	95% CI	p value
Département at-risk	2.57	0.86–10.1	0.094
Owned, no hunting	0.06	0–0.53	0.008
Owned, occasional hunting	0.46	0.16–1.47	0.181
Owned, frequent hunting	0.68	0.19–2.43	0.545

CI: confidence interval; OR: odds ratio.

## Discussion

These results indicate that a substantial proportion of cats in France have been exposed to H5 subtype influenza viruses – suggesting that viral circulation in peridomestic commensal avifauna may have been underestimated in previous studies [8]. Most of this exposure involved H5Nx viruses of clade 2.3.4.4b, while only a minority of cats had higher serological titres against an H5N3 LPAIV, representative of LPAIV strains endemic in France. Whether the seropositive cats ever showed clinical signs suggestive of HPAIV H5N1 infection (notably neurological and/or respiratory disorders) is unknown ­— although the questionnaire included a question on this, the answers were vague and inconsistent, with poorly described clinical signs, preventing us from interpreting the data. However, recent data from the United States suggest that clinical forms are not uncommon [9]. Also, we did not assess exposure to human seasonal influenza viruses, although cats are susceptible to these as well [10], as the aim of the study was to investigate exposure to H5Nx viruses via wild and domestic avifauna only. As they can be exposed to both human and animal influenza viruses, they may serve as mixing vessels for the emergence of human-adapted strains. However, no case of cat-to-human transmission involving an H5Nx virus has been evidenced to date — the only case described so far involved an H7N2 strain [11].

## Conclusion

From an epidemiological perspective, cats occupy a unique position at the interface between domestic and wild environments. We recommend raising awareness among veterinarians about the risk of HPAIV H5Nx infections in cats, as these infections may be more frequent than previously recognised. In cases of clinical suspicion—particularly when a cat shows signs compatible with influenza and the epidemiological context suggests possible exposure to HPAIV H5Nx—appropriate preventive measures should be implemented.

## Supplementary Data

499000 Supplement

## References

[1] Fusaro A Gonzales JL Kuiken T Mirinavičiūtė G Niqueux É Ståhl K Avian influenza overview December 2023-March 2024. EFSA J. 2024;22(3):e8754. 38550271 10.2903/j.efsa.2024.8754 PMC10977096

[2] Peacock TP Moncla L Dudas G VanInsberghe D Sukhova K Lloyd-Smith JO The global H5N1 influenza panzootic in mammals. Nature. 2025;637(8045):304-13. 10.1038/s41586-024-08054-z 39317240

[3] Domańska-Blicharz K Świętoń E Świątalska A Monne I Fusaro A Tarasiuk K Outbreak of highly pathogenic avian influenza A(H5N1) clade 2.3.4.4b virus in cats, Poland, June to July 2023. Euro Surveill. 2023;28(31):2300366. 10.2807/1560-7917.ES.2023.28.31.2300366 37535474 PMC10401911

[4] Duijvestijn MBHM Schuurman NNMP Vernooij JCM van Leeuwen MAJM van den Brand JMA Wagenaar JA Highly pathogenic avian influenza (HPAI) H5 virus exposure in domestic cats and rural stray cats, the Netherlands, October 2020 to June 2023. Euro Surveill. 2024;29(44):2400326. 10.2807/1560-7917.ES.2024.29.44.2400326 39484684 PMC11528901

[5] French Ministry of Agriculture. Influenza aviaire (IA) - Liste des communes en zone à risque de diffusion (ZRD) et en zone à risque particulier (ZRP). [Avian influenza- List of municipalities in areas at risk of spread and in areas at particular risk]. DGAL/SDSBEA/2023-651. Paris: Ministry of Agriculture, Agrifood, and Forestry; 2023. Available from: https://info.agriculture.gouv.fr/boagri/instruction-2023-651

[6] De Benedictis P Anderson TC Perez A Viale E Veggiato C Tiozzo Caenazzo S A diagnostic algorithm for detection of antibodies to influenza A viruses in dogs in Italy (2006-2008). J Vet Diagn Invest. 2010;22(6):914-20. 10.1177/104063871002200610 21088175

[7] Kittelberger R McFadden AMJ Hannah MJ Jenner J Bueno R Wait J Comparative evaluation of four competitive/blocking ELISAs for the detection of influenza A antibodies in horses. Vet Microbiol. 2011;148(2-4):377-83. 10.1016/j.vetmic.2010.08.014 20843619

[8] Le Gall-Ladevèze C Vollot B Hirschinger J Lèbre L Aaziz R Laroucau K Limited transmission of avian influenza viruses, avulaviruses, coronaviruses and Chlamydia sp. at the interface between wild birds and a free-range duck farm. Vet Res. 2025;56(1):36. 10.1186/s13567-025-01466-3 39923111 PMC11806813

[9] Burrough ER Magstadt DR Petersen B Timmermans SJ Gauger PC Zhang J Highly pathogenic avian influenza A(H5N1) Clade 2.3.4.4b Virus infection in domestic dairy cattle and cats, United States, 2024. Emerg Infect Dis. 2024;30(7):1335-43. 10.3201/eid3007.240508 38683888 PMC11210653

[10] Zhao S Schuurman N Tieke M Quist B Zwinkels S van Kuppeveld FJM Serological screening of influenza A virus antibodies in cats and dogs indicates frequent infection with different subtypes. J Clin Microbiol. 2020;58(11):e01689-20. 10.1128/JCM.01689-20 32878956 PMC7587082

[11] Belser JA Pulit-Penaloza JA Sun X Brock N Pappas C Creager HM A Novel A(H7N2) Influenza Virus Isolated from a Veterinarian Caring for Cats in a New York City Animal Shelter Causes Mild Disease and Transmits Poorly in the Ferret Model. J Virol. 2017;91(15):e00672-17. 10.1128/JVI.00672-17 28515300 PMC5512233

