# Supplementary material for: Cats as sentinels of mammal exposure to H5Nx avian influenza viruses: a seroprevalence study, France, December 2023 to January 2025
Source: Euro Surveill. 2025 Mar 27;30(12):2500189. doi: 10.2807/1560-7917.ES.2025.30.12.2500189 (PMC11951414; doi:10.2807/1560-7917.ES.2025.30.12.2500189)
Supplement: Supplement [file 25-00189_BESSIERE_Supplement.pdf]

Appendix 1: Information on animals included in the study. Animals were presented to their veterinarian for a variety of reasons unrelated to the study, ranging from a preventive medicine consultation to surgery.

This supplementary material is hosted by *Eurosurveillance* as supporting information alongside the article Cats as sentinels of mammalian exposure to H5Nx avian influenza viruses: a seroprevalence study, France, December 2023 to January 2025, on behalf of the authors, who remain responsible for the accuracy and appropriateness of the content. The same standards for ethics, copyright, attributions and permissions as for the article apply. Supplements are not edited by *Eurosurveillance* and the journal is not responsible for the maintenance of any links or email addresses provided therein.

N°ID	Date (sampling)	Department	Breed	Weight (kg)	Sex	Age (years)	ELISA H5 positive	H5N1 SN titre	H5N3 SN titre	Dpt at risk	Time spent outdoors	Stray cat	Hunting behavior
IA-001	9/12/2023	46	European	5	Male (neutered)	2	No			No	>12h	No	Frequent
IA-002	11/12/2023	46	European	5.5	Male	4	No			No	>12h	No	Occasional
IA-003	11/12/2023	46	European		Female (neutered)	7	No			No	>12h	No	Occasional
IA-004	12/12/2023	46	Chartreux	3.6		14	No			No	1-12h	No	Occasional
IA-005	12/12/2023	46	European	4	Female	4	No			No	>12h	No	Frequent
IA-006	12/12/2023	46	European	5	Male (neutered)	3.5	No			No	>12h	No	Occasional
IA-007	13/12/2023	46		6	Male	4.5	No			No		No	Occasional
IA-008	13/12/2023	46	European	4	Female (neutered)	6.5	No			No	>12h	No	Frequent
IA-009	14/12/2023	46	European	4.5	Female (neutered)	14.5	No			No	>12h	No	Occasional
IA-010	15/12/2023	46	European	3.8	Female (neutered)	9.5	No			No	>12h	No	Frequent
IA-011	26/1/2024	46	European	5.2	Male (neutered)	7.5	No			No	>12h	No	Frequent
IA-012	26/1/2024	46	European	5.2	Male	8.5	Yes	1/320	1/80	No	>12h	No	Frequent
IA-013	26/1/2024	46	European	5.6	Male (neutered)	11	No			No	>12h	No	Frequent
IA-014	26/1/2024	46	European	4.6	Male (neutered)	7.5	No			No		No	
IA-015	11/1/2024	46	European		Female	1.5	No			No	>12h	No	Frequent
IA-016	16/1/2024	46	European		Female (neutered)	7.5	No			No	>12h	No	Frequent
IA-017	4/1/2024	46	European		Female (neutered)	10	No			No	>12h	No	Frequent
IA-018	4/1/2024	46	European		Male (neutered)	4.5	No			No	>12h	No	Frequent
IA-019	27/1/2024	46	European	5.1	Male (neutered)	9.5	No			No	>12h	No	Frequent
IA-020	5/1/2024	46	European		Female (neutered)	1	No			No	>12h	No	Frequent
IA-021	26/1/2024	46	European	4	Male (neutered)	3	No			No	>12h	No	Frequent
IA-022	26/1/2024	46	European	5	Male (neutered)	6	No			No	>12h	No	Frequent
IA-023	26/1/2024	46	European	3.5	Male (neutered)	1.5	No			No	>12h	No	Frequent
IA-024	26/1/2024	46	European	3	Female	8.5	No			No	>12h	No	Frequent
IA-025	26/1/2024	46	European	3.5	Female (neutered)	4	No			No	>12h	No	Frequent
IA-026	19/1/2024	46	European	6	Male (neutered)	7	No			No	>12h	No	Frequent
IA-027	18/1/2024	65	European	2.7	Female (neutered)	15	No			Yes	1-12h	No	Occasional
IA-028	12/1/2024	65	European	3.1	Female	8	No			Yes	1-12h	No	Occasional
IA-029	15/1/2024	65	European	4.46	Female (neutered)	11	No			Yes	1-12h	No	Occasional
IA-030	16/1/2024	65	European		Female (neutered)	12	No			Yes	1-12h	No	Frequent
IA-031	16/1/2024	65	European	4.9	Male (neutered)	5	No			Yes	1-12h	No	Occasional
IA-032	17/1/2024	65	Main Coon	6.5	Male (neutered)	3	No			Yes	1-12h	No	Frequent
IA-033	17/1/2024	65	European	3.9	Male (neutered)	16	No			Yes	1-12h	No	Occasional
IA-034	18/1/2024	65	European	2.6	Female	0.5	No			Yes	>12h	No	Frequent
IA-035	19/1/2024	65	European	5.9	Male (neutered)	10	No			Yes	1-12h	No	Occasional
IA-036	19/1/2024	65	European	5.6	Male (neutered)	6	No			Yes	1-12h	No	Frequent
IA-037	22/1/2024	65	European	4.6	Male (neutered)	8	No			Yes	<1h	No	Occasional
IA-038	22/1/2024	65	European	4.6	Male (neutered)	11	No			Yes	1-12h	No	Frequent
IA-039	23/1/2024	65	European	3.3	Male	0.5	No			Yes	1-12h	No	Frequent
IA-040	23/1/2024	65	European	2.7	Female	1	No			Yes	>12h	No	Frequent
IA-041	23/1/2024	65	European	3.8	Male	1	No			Yes	>12h	No	Frequent
IA-042	23/1/2024	65	European	2.5	Female	0.5	No			Yes	>12h	No	Frequent
IA-043	23/1/2024	65	European	3.8	Male	20	No			Yes	<1h	No	Occasional
IA-044	2/1/2024	9	European	3	Female (neutered)	14	No			No	<1h	No	None
IA-045	2/1/2024	9	European	4.55	Female (neutered)	5	No			No	1-12h	No	Occasional
IA-046	9/1/2024	9	European	3.65	Female (neutered)	1	No			No	<1h	No	None
IA-047	9/1/2024	9	European		Female (neutered)	0.5	No			No	<1h	No	None
IA-048	12/1/2024	9	European	4	Female (neutered)	10.5	No			No	1-12h	No	Occasional
IA-049	30/1/2024	9	European	4.9	Male (neutered)	12	No			No	1-12h	No	Occasional
IA-050	30/1/2024	9	European		Male (neutered)	1	No			No	1-12h	No	Occasional
IA-051	30/1/2024	9			Male (neutered)	0.5	No			No	<1h	No	None
IA-052	13/2/2024	9	European	4.6	Male	0.5	No			No	1-12h	No	None
IA-053	13/2/2024	9	European	2.5	Female	0.5	No			No	1-12h	No	None
IA-054	14/2/2024	9	European	3.5	Female	0.5	No			No	1-12h	No	None
IA-055	14/2/2024	9	European	8	Male (neutered)	8	No			No	1-12h	No	None
IA-056	6/3/2024	5	Main Coon	5.45	Male	3	No			Yes	1-12h	No	Occasional
IA-057	14/2/2024	5	European			4	No			Yes	>12h	No	
IA-058	12/1/2024	5	European	5.2	Female (neutered)	14.5	No			Yes	1-12h	No	Occasional
IA-059	10/1/2024	5	European	6.2	Male (neutered)	8	No			Yes	1-12h	No	Frequent

IA-060	10/1/2024	5	European	5.35	Male (neutered)	14.5	No			Yes	>12h	No	Occasional
IA-061	9/1/2024	5	European	2.8	Female (neutered)	3	No			Yes	>12h	No	Frequent
IA-062	14/2/2024	5	European	4.015	Male (neutered)		No			Yes		No	
IA-063	28/2/2024	5	European	5.05	Male (neutered)	7.5	No			Yes	1-12h	No	Frequent
IA-064	22/2/2024	15	European	3.5	Male	1.5	No			No	>12h	No	Occasional
IA-065	20/2/2024	15	European	3.5	Male	1.5	No			No	1-12h	No	Frequent
IA-066	15/2/2024	15	European	3.1	Male	1	No			No	>12h	No	Frequent
IA-067	15/2/2024	15	European	4.2	Male	4	No			No	>12h	Yes	NA
IA-068	5/2/2024	15	European	2.9	Male	1	No			No	>12h	No	Frequent
IA-069	8/2/2024	15	European	3	Female	3	No			No	>12h	No	Frequent
IA-070	8/2/2024	15	European	3.5	Female	3	No			No	>12h	No	Frequent
IA-071	8/2/2024	15	European	3.6	Female	4	No			No	>12h	No	Frequent
IA-072	8/2/2024	15	European	2.7	Male	1	No			No	>12h	No	Frequent
IA-073	31/1/2024	15	European	3.5	Female (neutered)	9	No			No	1-12h	No	Occasional
IA-074	23/1/2024	15	European	3.32	Female (neutered)	7	No			No	<1h	No	None
IA-075	10/1/2024	15	European	3.8	Female (neutered)	7	No			No	<1h	No	None
IA-076	6/1/2024	15	European		Male (neutered)	5	No			No	1-12h	No	Frequent
IA-077	25/1/2024	85	European	4.14	Male (neutered)	6.5	No			Yes	1-12h	No	Frequent
IA-078	9/1/2024	85	European	4	Male	3	No			Yes	>12h	Yes	NA
IA-079	9/1/2024	85	European	3.5	Male	3	Yes	1/640	1/40	Yes	>12h	Yes	NA
IA-080	10/1/2024	85	European	3.5	Male	2	No			Yes	>12h	Yes	NA
IA-081	10/1/2024	85	European	6	Male	5	No			Yes	>12h	Yes	NA
IA-082	10/1/2024	85	European	2.5	Female	2	No			Yes	>12h	Yes	NA
IA-083	30/1/2024	85	European	2.9	Male	1	No			Yes	>12h	Yes	NA
IA-084	13/2/2024	85	European	5	Male	3	No			Yes	>12h	Yes	NA
IA-085	29/1/2024	85	European	3.16	Female	4	No			Yes	>12h	Yes	NA
IA-086	29/1/2024	85	European	3.065	Male	1	No			Yes	>12h	Yes	NA
IA-087	16/2/2024	85	European	2	Female (neutered)	1	No			Yes	>12h	Yes	NA
IA-088	14/1/2024	85	European		Female (neutered)	3	Yes	1/320	1/160	Yes	>12h	Yes	NA
IA-089	12/1/2024	85	European		Male	1	No			Yes	>12h	Yes	NA
IA-090	16/1/2024	85	European		Male	2	No			Yes	>12h	Yes	NA
IA-091	12/1/2024	85	European		Female	1	No			Yes	>12h	Yes	NA
IA-092	22/12/2023	3	European	3.4	Male (neutered)	12	No			No	1-12h	No	Occasional
IA-093	9/1/2024	3	European	3.2	Female (neutered)	2.5	No			No	>12h	No	Occasional
IA-094	9/1/2024	3	European	4.4	Male (neutered)	1.5	No			No	>12h	No	Occasional
IA-095	9/1/2024	3	European	5.4	Male (neutered)	4.5	No			No	>12h	No	
IA-096	10/1/2024	3	European	6.14	Male (neutered)	10	Yes	1/320	1/160	No	>12h	No	Frequent
IA-097	16/1/2024	3	European	5.25	Male (neutered)	2	No			No	>12h	No	
IA-098	29/1/2024	3	European	5		8	No			No	1-12h	No	Occasional
IA-099	30/1/2024	3	European	2.1	Female (neutered)	1.5	No			No	1-12h	No	Occasional
IA-100	2/2/2024	3	European	4	Male (neutered)	1	No			No	1-12h	No	Frequent
IA-101	8/2/2024	3	European	2.7	Female (neutered)	2	No			No	1-12h	No	Frequent
IA-102	8/2/2024	3		4.6	Female (neutered)	2	No			No	>12h	No	Occasional
IA-103	20/2/2024	3	European	3.8	Male (neutered)	1	No			No	1-12h	No	Frequent
IA-104	27/2/2024	3	European	2.5		1.5	No			No	>12h	No	Occasional
IA-105	7/3/2024	3	European	5.3		7	No			No	>12h	No	Frequent
IA-106	8/3/2024	3	European	3.6	Female (neutered)	1	No			No	>12h	No	Occasional
IA-107	25/3/2024	49			Male	0.5	No			Yes	<1h	No	None
IA-108	22/3/2024	49	European		Female	3	No			Yes	<1h	No	None
IA-109	19/3/2024	49	European		Male	2	No			Yes	<1h	No	None
IA-110	19/3/2024	49	European		Male	0.5	No			Yes	<1h	No	None
IA-111	19/3/2024	49	European		Male	0.5	No			Yes	<1h	No	None
IA-112	13/3/2024	49	European	4.2	Female (neutered)	11	No			Yes	<1h	No	Frequent
IA-113	12/3/2024	49	European	3.2	Male (neutered)	11	No			Yes	>12h	No	None
IA-114	19/2/2024	49	European	2.9	Female (neutered)	2	No			Yes	1-12h	No	None
IA-115	19/2/2024	49	European	4.4	Male (neutered)	12	No			Yes	1-12h	No	None
IA-116	13/2/2024	49	European	2.3	Male (neutered)	13	No			Yes	>12h	No	Frequent
IA-117	14/2/2024	49	European	3.2	Female (neutered)	3	No			Yes	1-12h	No	Frequent
IA-118	14/2/2024	49	European	5.3	Male (neutered)	4	No			Yes	1-12h	No	None
IA-119	13/3/2024	29	European	6	Male (neutered)	3	No			Yes	1-12h	No	Occasional
IA-120	16/3/2024	29	European		Male (neutered)	13	No			Yes	1-12h	No	Occasional

IA-121	19/3/2024	29	European	3.1	Female (neutered)	17	Yes	1/640	1/160	Yes	<1h	No	Occasional
IA-122	19/3/2024	29	European	3.15	Female (neutered)	11.5	No			Yes	1-12h	No	Occasional
IA-123	20/3/2024	29	European	3.2	Female (neutered)	15	No			Yes	>12h	No	None
IA-124	20/3/2024	29	European	4.15	Male (neutered)	16	No			Yes	1-12h	No	None
IA-125	21/3/2024	29	European	2.85	Female (neutered)	12	No			Yes	1-12h	No	Frequent
IA-126	22/3/2024	29	European	4.3	Male (neutered)	13	No			Yes	1-12h	No	Occasional
IA-127	22/3/2024	29	European	3.65	Male (neutered)	17	No			Yes	1-12h	No	None
IA-128	22/3/2024	29	European	3.6	Male (neutered)	2	No			Yes		No	
IA-129	27/3/2024	29	European	3.5	Male (neutered)	13	Yes	1/160	1/40	Yes	>12h	No	Occasional
IA-130	27/3/2024	29	European	2.85	Female (neutered)	14	No			Yes	1-12h	No	None
IA-131	27/3/2024	29	European	4.4	Male (neutered)	13	No			Yes	>12h	No	None
IA-132	2/4/2024	29	European	5	Male	12	No			Yes	1-12h	No	None
IA-133	3/4/2024	29	European	4.5	Male (neutered)	12	No			Yes	>12h	No	None
IA-134	29/2/2024	34	European	5.4	Male (neutered)	3	No			Yes	>12h	No	Occasional
IA-135	19/2/2024	34	European	4.3	Male (neutered)	10	No			Yes	>12h	No	Occasional
IA-136	19/2/2024	34	European	4.5	Male (neutered)	8	No			Yes	1-12h	No	Occasional
IA-137		34	European	3.96	Male	2	No			Yes	1-12h	No	Occasional
IA-138	9/2/2024	34	European		Female (neutered)	1	No			Yes	1-12h	No	Frequent
IA-139	3/2/2024	34	European	4.745	Male (neutered)	4.5	No			Yes	1-12h	No	Occasional
IA-140	19/1/2024	34	European	5	Male (neutered)	4	No			Yes	1-12h	No	Occasional
IA-141	30/1/2024	34	European	3.7	Female (neutered)	13	No			Yes	>12h	No	Occasional
IA-142	24/1/2024	34	European	7.5	Male (neutered)	13	No			Yes	<1h	No	None
IA-143	24/1/2024	34	European	6.13	Female (neutered)	11	No			Yes	1-12h	No	Frequent
IA-144	23/1/2024	34	European	4.1	Male (neutered)	12	No			Yes	1-12h	No	Occasional
IA-145	17/1/2024	34	European	6.2	Male (neutered)	5	No			Yes	>12h	No	Frequent
IA-146	17/1/2024	34	European	4.8	Male (neutered)	1.5	No			Yes	>12h	Yes	NA
IA-147	12/1/2024	34	European	2.7	Female (neutered)	1	No			Yes	>12h	No	Frequent
IA-148	12/1/2024	34	European	3.3	Female (neutered)	1	No			Yes	>12h	No	Frequent
IA-149	5/1/2024	34	Main Coon		Female (neutered)	3	No			Yes	1-12h	No	Occasional
IA-150	4/1/2024	34	European	2.7	Female (neutered)	8	No			Yes	>12h	No	Frequent
IA-151	4/1/2024	34	European		Male (neutered)	1.5	No			Yes	>12h	No	Occasional
IA-152	12/2/2024	43	European	5	Male	4	No			No	>12h	No	Occasional
IA-153	14/2/2024	43	European	5.6	Male	5	No			No	>12h	No	Frequent
IA-154	15/2/2024	43	Oriental	4.92	Male (neutered)	8	No			No	1-12h	No	Occasional
IA-155	19/2/2024	43	European	3.8	Female (neutered)	4	No			No	1-12h	No	Occasional
IA-156	20/2/2024	43	European	4.5	Male (neutered)	2.5	No			No	>12h	No	Occasional
IA-157	23/2/2024	43	European		Male (neutered)	9	No			No	1-12h	No	Occasional
IA-158	24/2/2024	43	European	4.7	Female (neutered)	9	No			No	>12h	No	Occasional
IA-159	27/2/2024	43	European	3.9	Female (neutered)	9	No			No	1-12h	No	Occasional
IA-160	4/3/2024	43	European	4	Female (neutered)	2.5	No			No	>12h	No	Occasional
IA-161	6/3/2024	43	European	4.8	Male (neutered)	4	No			No	>12h	No	Occasional
IA-162	7/3/2024	43	European	3.37	Female (neutered)	1.5	No			No	>12h	No	Occasional
IA-163	11/3/2024	43		6.4	Male (neutered)	14	No			No	1-12h	No	None
IA-164	11/3/2024	43	European	4	Male (neutered)	1.5	No			No	1-12h	No	Occasional
IA-165	27/3/2024	43	European	5.5	Female	8	No			No	1-12h	No	
IA-166	8/4/2024	43	European	5.4	Female (neutered)	6.5	No			No	>12h	No	Occasional
IA-167	4/4/2024	44	European	3.85	Female	1	No			Yes	1-12h	No	Occasional
IA-168	19/3/2024	44	European	3	Female	1	No			Yes	1-12h	No	Occasional
IA-169	19/3/2024	44	European	3.58	Female	1	No			Yes	1-12h	No	Occasional
IA-170	19/3/2024	44	European	6	Male	10	No			Yes	>12h	Yes	NA
IA-171	21/2/2024	44	European	4.3	Female	2	No			Yes	1-12h	No	Occasional
IA-172	14/2/2024	44	European	3.5	Female (neutered)	1.5	No			Yes	>12h	Yes	NA
IA-173	10/1/2024	44	European	3.8	Male (neutered)	3	No			Yes	>12h	No	Frequent
IA-174	10/1/2024	44	European	3.8	Male (neutered)	3	No			Yes	>12h	No	Frequent
IA-175	26/3/2024	44	European	3.39	Female	5	No			Yes	1-12h	No	Occasional
IA-176	27/3/2024	44	European	4.37	Male (neutered)	10	Yes	1/320	1/80	Yes	1-12h	No	Occasional
IA-177	4/4/2024	44	European	4	Female	1	No			Yes	>12h	No	Occasional
IA-178	2/2/2024	35	European		Female (neutered)		No			Yes	>12h	No	Occasional
IA-179	9/2/2024	35	European		Male (neutered)	12	No			Yes	1-12h	No	Occasional
IA-180	9/2/2024	35	European	3.6	Female (neutered)	1.8	No			Yes	1-12h	No	Occasional
IA-181	9/2/2024	35	European	4.275	Female (neutered)	1.9	No			Yes	1-12h	No	Occasional

IA-182	12/2/2024	35	European	3.22	Male (neutered)	14.6	No			Yes	1-12h	No	Frequent
IA-183	19/2/2024	35	European	7.15	Male (neutered)	12.75	No			Yes	>12h	No	Frequent
IA-184	19/2/2024	35	European	4.3	Female (neutered)	13.3	No			Yes	1-12h	No	
IA-185	20/2/2024	35	European	5.11	Male (neutered)	13.3	No			Yes	1-12h	No	None
IA-186	20/2/2024	35	European	5.43	Female (neutered)	12.5	No			Yes	1-12h	No	Occasional
IA-187	27/2/2024	35	European	3.85	Male (neutered)	9.75	No			Yes	1-12h	No	Occasional
IA-188	26/3/2024	35	European	3.75	Male (neutered)	2	No			Yes	>12h	No	Frequent
IA-189	27/3/2024	35	Norvegian	4.83	Male (neutered)	11	No			Yes	1-12h	No	None
IA-190	2/4/2024	35	European	7.07	Male (neutered)	14.7	No			Yes	1-12h	No	Occasional
IA-191	9/4/2024	35	European	4.6	Female (neutered)	6	No			Yes	1-12h	No	Occasional
IA-192		35	European	6.23	Male (neutered)	13	No			Yes	<1h	No	None
IA-193	5/3/2024	50	European	5.1	Female (neutered)	8.5	No			Yes	1-12h	No	None
IA-194	5/3/2024	50	European	4.4	Female (neutered)	2.5	No			Yes	1-12h	No	Occasional
IA-195	5/3/2024	50	European	3.7	Female (neutered)	3.5	No			Yes	1-12h	No	Occasional
IA-196	11/3/2024	50	European		Female (neutered)	1	No			Yes	>12h	Yes	NA
IA-197	12/3/2024	50	European		Male	1	No			Yes	>12h	Yes	NA
IA-198	12/3/2024	50	European	4.6	Female (neutered)	14	No			Yes	<1h	No	None
IA-199		50					No			Yes	1-12h	No	Occasional
IA-200	14/3/2024	50	European	5.4	Male (neutered)	12.5	No			Yes	<1h	No	Occasional
IA-201		50	European		Female (neutered)	12	No			Yes	1-12h	No	None
IA-202	19/3/2024	50	European		Female (neutered)	1	No			Yes	>12h	No	Frequent
IA-203	21/3/2024	50	European	3.25	Female (neutered)	14.5	No			Yes	1-12h	No	None
IA-204	25/3/2024	50	European	3.5	Female (neutered)	1	No			Yes	1-12h	No	Occasional
IA-205	25/3/2024	50	European	6.4	Male (neutered)	10	No			Yes	1-12h	No	Occasional
IA-206	26/3/2024	50	European	3.1	Female (neutered)	3	No			Yes	>12h	No	Occasional
IA-207	2/4/2024	50	European	4.5	Male (neutered)	3	No			Yes	1-12h	No	Occasional
IA-208	23/2/2024	51	European	3.75	Female (neutered)	2.5	No			Yes	1-12h	No	Occasional
IA-209	15/4/2024	51	Ragdoll	6	Female (neutered)	10	No			Yes	1-12h	No	Occasional
IA-210	29/4/2024	51	European	4.3	Male (neutered)	1	No			Yes	1-12h	No	Occasional
IA-211	30/4/2024	51	European	5	Female (neutered)	13	No			Yes	1-12h	No	Occasional
IA-212	30/4/2024	51	European	3.9	Female (neutered)	14	No			Yes	1-12h	No	Occasional
IA-213	2/5/2024	51	European	3.1	Female (neutered)	9	No			Yes	<1h	No	Occasional
IA-214	2/5/2024	51	European	4.7	Female (neutered)	5	No			Yes	1-12h	No	Frequent
IA-215	3/5/2024	51	European	5.8	Male (neutered)	4	No			Yes	1-12h	No	Frequent
IA-216	3/5/2024	51	European	5		14	No			Yes	1-12h	No	Occasional
IA-217	6/5/2024	51	European	3.6	Male (neutered)	1	No			Yes	1-12h	No	Frequent
IA-218	5/2/2024	53	European	5.3	Male (neutered)	6.5	No			Yes	1-12h	No	Occasional
IA-219	5/2/2024	53	European	3.3	Male	2.5	No			Yes	<1h	No	None
IA-220	9/2/2024	53	European	3.8	Male (neutered)	8	No			Yes	1-12h	No	Occasional
IA-221	12/2/2024	53	European	3.95	Male	4	No			Yes	>12h	No	Frequent
IA-222	13/2/2024	53	European	6	Male	9	No			Yes	1-12h	No	Occasional
IA-223	13/2/2024	53	European		Male	2.7	No			Yes	1-12h	No	
IA-224	19/2/2024	53	European	4.5	Male	4	No			Yes	1-12h	No	Occasional
IA-225	22/2/2024	53	Oriental	5.5	Male	10.5	No			Yes		No	
IA-226	16/6/2026	53	European	4	Male	9	No			Yes	1-12h	No	Frequent
IA-227	17/6/2024	53	European	3.3	Female	8	No			Yes	1-12h	No	Occasional
IA-228	12/7/2024	65	European	3.5	Female	3	No			Yes	1-12h	No	Occasional
IA-229	11/7/2024	65	European		Female	3	No			Yes	>12h	Yes	NA
IA-230	4/7/2024	65	European	3	Female (neutered)	1	No			Yes	>12h	No	Frequent
IA-231	4/7/2024	65	European	2.9	Female (neutered)	2	No			Yes	>12h	No	Occasional
IA-232	3/7/2024	65	European		Male		No			Yes	1-12h	No	Occasional
IA-233	2/7/2024	65	European	3	Female	0.5	No			Yes	>12h	No	Occasional
IA-234	1/7/2024	65	European	5.7	Male (neutered)	3	No			Yes	1-12h	No	Frequent
IA-235	29/6/2024	65	European	4.6	Male (neutered)	1	Yes	1/640	1/40	Yes	>12h	No	Frequent
IA-236	25/6/2024	65	European	5.3	Female (neutered)	13	No			Yes	<1h	No	None
IA-237	24/6/2024	65	European	4.5	Male (neutered)	1	No			Yes	1-12h	No	Occasional
IA-238	12/6/2024	65	European		Female (neutered)		No			Yes	<1h	No	None
IA-239	10/6/2024	65	European	3.5	Female (neutered)	13	No			Yes	>12h	No	Frequent
IA-240	5/6/2024	65	European	2.9	Female (neutered)		No			Yes	>12h	No	Frequent
IA-241	4/6/2024	65	European	4	Female (neutered)	6.5	No			Yes	1-12h	No	Occasional
IA-242	6/6/2024	65	European	5.9	Male (neutered)	2	No			Yes	1-12h	No	Occasional

IA-243	19/6/2024	65	European	2.9	Male	0.5	No			Yes	1-12h	No	Occasional
IA-244	4/6/2024	65	European	2.6	Female	1	No			Yes	>12h	No	Frequent
IA-245	5/7/2024	65	European	4.7	Male	7	No			Yes	>12h	No	Frequent
IA-246	10/7/2024	65	European	3	Female	1.5	No			Yes	>12h	No	Occasional
IA-247	30/4/2024	12	European	4.6	Female (neutered)	13	No			No	<1h	No	Occasional
IA-248	28/2/2024	12	European	5.3	Male (neutered)	5.5	No			No	1-12h	No	Frequent
IA-249	26/2/2024	12	European	3	Female	2.5	No			No	>12h	Yes	NA
IA-250	30/4/2024	12	European	3.7	Male (neutered)	17	No			No	1-12h	No	Occasional
IA-251	26/2/2024	12	European			13.5	No			No	1-12h	No	Occasional
IA-252	28/3/2024	12	European				No			No	>12h	No	Frequent
IA-253	3/7/2024	59	European	4.5	Male (neutered)	7	No			Yes	>12h	No	Occasional
IA-254	28/6/2024	59	European	4.8	Male (neutered)	12	No			Yes	>12h	No	Occasional
IA-255	14/6/2024	59	European	4.3	Male	12	No			Yes	1-12h	No	None
IA-256	14/6/2024	59			Female	15	No			Yes	1-12h	No	None
IA-257	8/4/2024	59	European	3.2	Male (neutered)	1.5	No			Yes	1-12h	No	Occasional
IA-258	17/5/2024	59	European	4	Male (neutered)	12	No			Yes	>12h	No	Frequent
IA-259	18/5/2024	59	European	5	Male (neutered)	13	No			Yes	<1h	No	None
IA-260	15/5/2024	59	European	3.6	Female	2	No			Yes	>12h	No	
IA-261	20/3/2024	59	European	4.7		11	No			Yes	1-12h	No	Occasional
IA-262	20/3/2024	59	European	5	Male (neutered)	10	Yes	1/320	1/160	Yes	1-12h	No	Occasional
IA-263	13/3/2024	59	European	5	Male (neutered)	3.5	No			Yes	1-12h	No	Occasional
IA-264	21/2/2024	59	Norvegian	7	Male (neutered)	5	No			Yes	1-12h	No	Frequent
IA-265	28/6/2024	59				16	No			Yes	>12h	No	Occasional
IA-266	19/7/2024	43	European	6	Male	9	No			No	1-12h	No	None
IA-267	10/7/2024	43		4.3	Male	1	No			No	>12h	No	Occasional
IA-268	8/7/2024	43	European	3.6	Female (neutered)	9	No			No	1-12h	No	
IA-269	5/7/2024	43	European		Female	1.5	No			No	>12h	No	Occasional
IA-270	24/6/2024	43	European	6.5		7	No			No	1-12h	No	None
IA-271	29/4/2024	43	European	7	Male (neutered)	4	No			No	1-12h	No	Occasional
IA-272	30/5/2024	43	European	4	Female	5	No			No	>12h	No	
IA-273	31/5/2024	43	European	3.4	Male (neutered)	1	No			No	>12h	No	Occasional
IA-274	7/6/2024	43	European	3.2	Female	1	No			No	1-12h	No	Occasional
IA-275	19/4/2024	37	Main Coon	8	Male (neutered)	1.5	No			Yes	1-12h	No	None
IA-276	22/4/2024	37	European	2.9	Female (neutered)	11.5	No			Yes	1-12h	No	Occasional
IA-277	22/4/2024	37	European	4.1	Male (neutered)	7.5	No			Yes	1-12h	No	Occasional
IA-278	23/4/2024	37	European	3.8	Female (neutered)	2	No			Yes	>12h	No	Occasional
IA-279	6/5/2024	37	European	3	Female (neutered)		No			Yes	>12h	No	
IA-280	20/5/2024	37	European	3.4	Female (neutered)	2	No			Yes	>12h	Yes	NA
IA-281	21/5/2024	37	European	3	Male (neutered)	1.5	No			Yes	1-12h	No	Occasional
IA-282	16/7/2024	37	European	3.2	Female (neutered)	1	No			Yes	>12h	Yes	NA
IA-283	16/7/2024	37	European	4.2	Female (neutered)	1	No			Yes	>12h	Yes	NA
IA-284	16/7/2024	37	European	4	Male (neutered)	1	No			Yes	>12h	Yes	NA
IA-285	16/7/2024	37	European	3.4	Male (neutered)	1	No			Yes	>12h	Yes	NA
IA-286	30/1/2024	32	European	3	Female	1	No			Yes	>12h	Yes	NA
IA-287	30/1/2024	32	European	3		2	No			Yes	>12h	Yes	NA
IA-288	30/1/2024	32	European	3	Male	1	No			Yes	>12h	Yes	NA
IA-289	30/1/2024	32	European	4	Male	1	No			Yes	>12h	Yes	NA
IA-290	30/1/2024	32	European	1.5	Male	0.5	No			Yes	>12h	Yes	NA
IA-291	30/1/2024	32	European	5	Male	5.5	No			Yes	>12h	Yes	NA
IA-292	1/2/2024	32	European		Male	12	No			Yes	1-12h	No	Occasional
IA-293	1/2/2024	32	European	3	Female	1	No			Yes	1-12h	No	Occasional
IA-294	1/2/2024	32	European	2.8	Female	11.5	No			Yes	1-12h	No	Occasional
IA-295	20/6/2024	32	European		Female	2	No			Yes	>12h	Yes	NA
IA-296	20/6/2024	32	European		Male	2	No			Yes	>12h	Yes	NA
IA-297	11/6/2024	64	European	4.84	Male	0.5	No			Yes	>12h	No	Frequent
IA-298	6/6/2024	64	European	3.9	Female (neutered)	5.5	No			Yes	1-12h	No	Occasional
IA-299	2/7/2024	64	European	2.92	Female (neutered)	13.5	No			Yes	1-12h	No	None
IA-300	2/7/2024	64	European	3.75	Female (neutered)	11	No			Yes	1-12h	No	Occasional
IA-301	1/7/2024	64	European	4.85	Female (neutered)	14.5	No			Yes	1-12h	No	Occasional
IA-302	6/6/2024	64	European	3.49	Female (neutered)	1	No			Yes	1-12h	No	Occasional
IA-303	30/5/2024	64	European	3.12	Female (neutered)	14	No			Yes	1-12h	No	None

IA-304	7/6/2024	64	European	4.15	Female (neutered)	8	No			Yes	1-12h	No	Occasional
IA-305	31/5/2024	64	European	5.32	Female (neutered)	14	No			Yes	1-12h	No	None
IA-306	14/6/2024	64	European	4.2	Male (neutered)	4	No			Yes	>12h	No	Occasional
IA-307	27/6/2024	64	European	4.6	Female (neutered)	2	No			Yes	>12h	Yes	NA
IA-308	2/7/2024	64	European	3.7	Female (neutered)	11	No			Yes	1-12h	No	Occasional
IA-309	2/7/2024	64	European	4	Female (neutered)	5	No			Yes	>12h	Yes	NA
IA-310	9/7/2024	64	European	4.7	Male (neutered)	12	No			Yes	1-12h	No	Occasional
IA-311	26/6/2024	64	European				No			Yes	1-12h	No	Occasional
IA-312	30/5/2024	64	Main Coon	6.8	Male (neutered)	8	No			Yes	1-12h	No	None
IA-313	1/6/2024	64	European	6	Male (neutered)	12	No			Yes	1-12h	No	Occasional
IA-314	10/6/2024	64	European	4.2	Female (neutered)	6	No			Yes	>12h	No	Occasional
IA-315	19/7/2024	64	European		Male (neutered)	3	No			Yes	1-12h	No	Occasional
IA-316	19/7/2024	64	European		Male (neutered)	17	No			Yes	1-12h	No	Occasional
IA-317	20/2/2024	83	European	4.7	Female	5	No			Yes	>12h	No	Occasional
IA-318	21/2/2024	83	European	5.4	Male (neutered)	10	No			Yes		No	
IA-319	28/2/2024	83	European		Male (neutered)	12	No			Yes	>12h	No	Occasional
IA-320	15/4/2024	83	European	3.6	Female (neutered)	13	No			Yes	1-12h	No	None
IA-321	17/4/2024	83	European	5.5	Male (neutered)	10	No			Yes	<1h	No	None
IA-322	18/4/2024	83	Persan	4		12	No			Yes	1-12h	No	Frequent
IA-323	15/7/2024	83	European	5	Male (neutered)	5	No			Yes	1-12h	No	Occasional
IA-324	16/7/2024	83	European	3.7	Male (neutered)	12	No			Yes	1-12h	No	Occasional
IA-325	24/7/2024	83	European	5.85	Female (neutered)	8	No			Yes	1-12h	No	Occasional
IA-326	25/7/2024	83	European	4.2	Male	6	No			Yes	>12h	No	Frequent
IA-327	29/7/2024	44	European	2.9	Male (neutered)	5	No			Yes	>12h	No	Occasional
IA-328	25/7/2024	44	European	4.44	Male (neutered)	8	No			Yes	>12h	No	Frequent
IA-329	26/6/2024	44	European	2.8	Female	1.5	No			Yes	>12h	Yes	NA
IA-330	26/7/2024	44	European	3.3	Female	1	No			Yes	1-12h	No	Occasional
IA-331	31/5/2024	44	European	3	Female (neutered)	1	No			Yes	>12h	Yes	NA
IA-332	25/7/2024	44	European	4.3	Male (neutered)	4	No			Yes	>12h	No	Frequent
IA-333	6/6/2024	44		3.7	Male	7	No			Yes	>12h	Yes	NA
IA-334	26/7/2024	44	European	5	Male (neutered)	12	No			Yes	1-12h	No	Occasional
IA-335	16/7/2024	44	European	2.8	Female	1.5	Yes	1/640	1/160	Yes	>12h	Yes	NA
IA-336	23/7/2024	44	European	2.71	Female	1	No			Yes	>12h	Yes	NA
IA-337	26/7/2024	44	European	4.98	Female (neutered)	16	No			Yes	1-12h	No	Occasional
IA-338		60					No			No		No	
IA-339	3/7/2024	60	European	4	Female (neutered)	8	No			No	<1h	No	Occasional
IA-340	18/4/2024	60	European	2.475	Female	0.6	No			No		No	
IA-341	14/5/2024	60	European	3.2	Female (neutered)	1	No			No	<1h	No	None
IA-342	25/6/2024	60	European	4.6	Female (neutered)	3	No			No		No	Occasional
IA-343	2/7/2024	60	European	3.6	Male	1.5	No			No		No	
IA-344	23/4/2024	60	European	3.265	Male	0.7	No			No	<1h	No	Occasional
IA-345		60					No			No		No	
IA-346	23/4/2024	60	European		Female	1	No			No	1-12h	No	Frequent
IA-347	13/6/2024	60	European	4	Male (neutered)	0.7	No			No	1-12h	No	Occasional
IA-348	16/5/2024	60	European	2.615	Female (neutered)	0.11	No			No	>12h	Yes	NA
IA-349	3/6/2024	60	European	3	Female (neutered)	0.7	No			No	<1h	No	Occasional
IA-350	30/4/2024	40	European	4.1	Female (neutered)	10.5	No			Yes	1-12h	No	Occasional
IA-351	3/5/2024	40	European	5	Female (neutered)	14.5	No			Yes		No	
IA-352	30/5/2024	40	European	3.15	Male	14	No			Yes	1-12h	No	None
IA-353	26/4/2024	40	European		Male (neutered)	10	No			Yes	1-12h	No	
IA-354	31/5/2024	40	European	3.3	Female	6	No			Yes	<1h	No	None
IA-355	31/5/2024	40	European	3.95	Male	0.6	No			Yes	<1h	No	None
IA-356	31/5/2024	40	European	2.6	Female	1	No			Yes		No	
IA-357	31/5/2024	40	European	3.34	Female (neutered)	16.5	No			Yes	1-12h	No	
IA-358	31/5/2024	40	European	4.36	Male	2	No			Yes	1-12h	No	
IA-359	31/5/2024	40	European	5.09	Male (neutered)	11.5	No			Yes		No	
IA-360	1/6/2024	40	European	5	Male (neutered)	8	No			Yes	1-12h	No	Occasional
IA-361	4/6/2024	40	European	3.1	Female	2	No			Yes	1-12h	No	
IA-362	4/6/2024	40	European	3.85	Female (neutered)	6	No			Yes	1-12h	No	
IA-363	7/6/2024	40	Main Coon	5.1	Male (neutered)	0.8	No			Yes	1-12h	No	
IA-364	7/6/2024	40	Main Coon	3.4	Female	1.3	No			Yes	1-12h	No	

IA-365	7/6/2024	40	Oriental	3.48	Male (neutered)	16	Yes	0	1/40	Yes	<1h	No	
IA-366	8/6/2024	40	European	2.94	Female (neutered)	16	No			Yes	1-12h	No	
IA-367	11/6/2024	40	European	5.8	Male	1	No			Yes	1-12h	No	
IA-368	11/6/2024	40	European	3.5	Female	1	No			Yes	1-12h	No	Occasional
IA-369	11/6/2024	40	European	3	Female	2	No			Yes	1-12h	No	Occasional
IA-370	28/6/2024	40	Main Coon	7.96	Male (neutered)	1.5	No			Yes	1-12h	No	
IA-371	28/6/2024	40	European	5.6	Male (neutered)	1.5	No			Yes	1-12h	No	
IA-372	2/7/2024	40	European	3.65	Female (neutered)	13	No			Yes	1-12h	No	
IA-373	4/7/2024	40	European	4.6	Female (neutered)	11	No			Yes	1-12h	No	
IA-374	5/7/2024	40	European	2.34	Female	1	No			Yes	1-12h	No	Occasional
IA-375	5/7/2024	40	European	3.5	Female	1	No			Yes	1-12h	No	
IA-376	5/7/2024	46	European	4.7	Male (neutered)	16	No			No	1-12h	No	Occasional
IA-377	15/7/2024	46			Male	1	No			No	>12h	No	Frequent
IA-378	16/7/2024	46	European	3.5	Female (neutered)	1	No			No	<1h	No	Frequent
IA-379	16/7/2024	46	European	4	Female (neutered)	1	No			No	>12h	No	Frequent
IA-380	16/7/2024	46	European		Male (neutered)	3	No			No	>12h	No	Frequent
IA-381	16/7/2024	46	European	3.4	Female	1	No			No	>12h	No	Frequent
IA-382	6/7/2024	46	European	6	Male (neutered)	5	No			No	>12h	No	Frequent
IA-383	12/7/2024	46	European	4.5	Male	2	No			No	>12h	No	Occasional
IA-384	3/8/2024	46	European	3	Female (neutered)	5	No			No	>12h	No	Frequent
IA-385	21/7/2024	46	European	5.6	Female (neutered)	13	No			No	>12h	No	Frequent
IA-386	21/7/2024	46	European	4	Female	9	No			No	1-12h	No	Frequent
IA-387	22/7/2024	46	European	3	Female (neutered)	14	No			No	1-12h	No	Occasional
IA-388	22/07/204	46	European		Male (neutered)	1	No			No	1-12h	No	Frequent
IA-389	22/7/2024	46	Ragdoll		Female (neutered)	12	No			No	<1h	No	Occasional
IA-390	23/7/2024	46	Russian blue (	7	Male (neutered)	10	No			No	1-12h	No	Occasional
IA-391	23/7/2024	46	European	2.5	Female (neutered)	14	No			No	1-12h	No	Frequent
IA-392	24/7/2024	46	European	4	Female (neutered)	16	No			No	1-12h	No	Frequent
IA-393	24/7/2024	46	European		Female (neutered)	11	No			No	1-12h	No	Occasional
IA-394	27/7/2024	46	European		Male (neutered)	15	No			No	>12h	No	Frequent
IA-395	29/7/2024	46	European	4.1	Male (neutered)	16	No			No	1-12h	No	Occasional
IA-396	31/7/2024	46	European		Female (neutered)	5	No			No	1-12h	No	Frequent
IA-397	1/8/2024	46	European	3.6	Female (neutered)	13	No			No	1-12h	No	Occasional
IA-398	3/8/2024	46	European	4.2	Male (neutered)	10	No			No	>12h	No	Frequent
IA-399	3/8/2024	46		3.35	Male	9	No			No	1-12h	No	Occasional
IA-400	5/8/2024	46	European	3.5	Female (neutered)	3	No			No	>12h	No	Frequent
IA-401	5/8/2024	46	European	6.3	Male	15	No			No	>12h	No	Frequent
IA-402	5/8/2024	46	European	5.4	Female	7	No			No	>12h	No	Frequent
IA-403	7/8/2024	46	European	7.1	Female (neutered)	12	No			No	1-12h	No	Occasional
IA-404	8/8/2024	46	European	3.8	Female	6	No			No	>12h	No	Frequent
IA-405	7/8/2024	46	Chartreux		Male	1	No			No	1-12h	No	Frequent
IA-406	8/8/2024	46	European		Female	14	No			No	1-12h	No	Occasional
IA-407	9/8/2024	46	European	4.2	Male (neutered)	4	No			No	1-12h	No	Frequent
IA-408	9/8/2024	46	European	4	Male	2	No			No	>12h	No	Frequent
IA-409	9/8/2024	46	European	3.75	Male	2	No			No	1-12h	No	Occasional
IA-410	17/8/2024	46	European	7.1	Male (neutered)	14	No			No	1-12h	No	Occasional
IA-411	19/8/2024	46	European		Female (neutered)	14	No			No	1-12h	No	Occasional
IA-412	19/8/2024	46	European		Female (neutered)	12	No			No	1-12h	No	Occasional
IA-413	20/8/2024	46	European	2.5	Female (neutered)	10	No			No	1-12h	No	Occasional
IA-414	20/8/2024	46	European	5.3	Male (neutered)	13	No			No	>12h	No	Frequent
IA-415	21/8/2024	46	European	6.35	Male (neutered)	15	No			No	1-12h	No	Occasional
IA-416	21/8/2024	46	European	3.8	Male	10	No			No	1-12h	No	Occasional
IA-417	22/8/2024	46	European	5.7	Female (neutered)	9	No			No	>12h	No	Frequent
IA-418	22/8/2024	46	European	3.15	Female	8	No			No	1-12h	No	Occasional
IA-419	23/8/2024	46	European	5.3	Male (neutered)	4	No			No	1-12h	No	Frequent
IA-420	23/8/2024	46	European	3	Female (neutered)	11	No			No	1-12h	No	Occasional
IA-421		46					No			No		No	
IA-422	15/3/2024	25	European	3.8	Male	11.9	No			Yes	1-12h	No	Occasional
IA-423	19/3/2024	25	European	3.7	Female (neutered)	5	No			Yes	>12h	Yes	NA
IA-424	26/3/2024	25	European	4.3	Male	5	No			Yes	>12h	Yes	NA
IA-425	9/4/2024	25	European	3.25	Male	2	No			Yes	1-12h	No	Occasional

IA-426	15/5/2024	25	European	6.8	Female (neutered)	9	No			Yes	<1h	No	None
IA-427	16/7/2024	25	European	3.2	Female (neutered)	8	No			Yes	1-12h	No	
IA-428	16/7/2024	25	European	5.1	Male (neutered)	2	No			Yes	1-12h	No	
IA-429	23/2/2024	25	European	4.5	Male (neutered)	5	No			Yes	>12h	No	Frequent
IA-430	24/2/2024	25	European	4.1	Female (neutered)	11	No			Yes	1-12h	No	Occasional
IA-431	8/3/2024	25			Male	7	No			Yes	>12h	No	
IA-432	8/3/2024	25	European	5.9	Male (neutered)	6	No			Yes	1-12h	No	Occasional
IA-433	14/3/2024	25	European	5.4	Male (neutered)	2.11	No			Yes	1-12h	No	None
IA-434	16/8/2024	21	European	4.7	Female (neutered)	5	No			Yes	1-12h	No	Occasional
IA-435	16/8/2024	21	European	4.7	Female (neutered)	10	No			Yes	<1h	No	None
IA-436	23/7/2024	21	European	2.15	Female (neutered)	12	No			Yes	1-12h	No	None
IA-437	6/8/2024	21	European	2.65	Female (neutered)	12	No			Yes	>12h	No	Occasional
IA-438	6/8/2024	21	European	5.2	Male (neutered)	3	No			Yes	<1h	No	None
IA-439	1/8/2024	21	European	2.7	Male (neutered)	18	No			Yes	<1h	No	None
IA-440	19/7/2024	21	Siamese (cros	2.5	Male (neutered)	11	No			Yes	<1h	No	None
IA-441	19/7/2024	21	European	3.4	Male (neutered)	14	No			Yes	1-12h	No	None
IA-442	29/7/2024	21	European	3.16	Female (neutered)	14	No			Yes		No	
IA-443	12/8/2024	21	European	4.5	Male (neutered)	14	No			Yes	1-12h	No	Occasional
IA-444	27/8/2024	17	European	2.6	Female	1	No			Yes	>12h	Yes	NA
IA-445	7/3/2024	17	European	4	Male	3	No			Yes	1-12h	No	Occasional
IA-446	20/5/2024	17	European	4.8	Male (neutered)	3	No			Yes	1-12h	No	Occasional
IA-447	11/3/2024	17	European	3.2	Female	1	No			Yes	>12h	Yes	NA
IA-448	8/5/2024	17	European	3	Female (neutered)	1	No			Yes	1-12h	No	
IA-449	17/4/2024	17	European	4.1	Female (neutered)	12	No			Yes		No	
IA-450	14/3/2024	17	European	2.9	Female	1	No			Yes	>12h	Yes	NA
IA-451	7/3/2024	17	European	4.5	Male (neutered)	3	No			Yes	>12h	Yes	NA
IA-452	26/3/2024	17	European	3.2	Female	1	No			Yes	>12h	Yes	NA
IA-453	10/7/2024	31	European	4.12	Male (neutered)	0.9	No			No	1-12h	No	None
IA-454	10/7/2024	31	European	4.05	Female (neutered)	0.7	No			No	intérieur stric	No	None
IA-455	10/7/2024	31	European	2.54	Female (neutered)	2.1	Yes	1/320	1/80	No	>12h	No	Occasional
IA-456	10/7/2024	31	European	6	Male (neutered)	1.5	No			No	>12h	No	Frequent
IA-457	16/7/2024	31	European	5.02	Female (neutered)	11	No			No	1-12h	No	Frequent
IA-458	16/7/2024	31	European	3.95	Male (neutered)	12	No			No	1-12h	No	Occasional
IA-459	9/8/2024	31	European	4.05	Male (neutered)	5	No			No	1-12h	No	Occasional
IA-460	26/8/2024	31	European	4.34		13	No			No	1-12h	No	Occasional
IA-461	27/8/2024	31	European	4.7	Female (neutered)	7	No			No	>12h	No	Occasional
IA-462	31/7/2024	31				8	No			No		No	
IA-463	21/8/2024	65	European	4	Male (neutered)	1.5	No			Yes	>12h	No	
IA-464	16/7/2024	65	European	5.5	Male (neutered)	8	No			Yes	>12h	No	
IA-465	6/7/2024	65	European	4.2	Male	3	No			Yes	>12h	Yes	NA
IA-466	3/7/2024	65	European	7.1	Male (neutered)	3.5	No			Yes	1-12h	No	
IA-467	2/7/2024	65	European	2.9	Female	1.5	No			Yes	>12h	No	
IA-468	2/7/2024	65	European	4.3	Male	1	No			Yes	>12h	No	
IA-469	27/6/2024	65	European	3.7	Male	2	No			Yes	1-12h	No	
IA-470	26/6/2024	65	European	5	Male (neutered)	11	No			Yes	1-12h	No	
IA-471	25/6/2024	65		5.3	Female	13	No			Yes	>12h	No	
IA-472	25/6/2024	65	European	3	Female	1	No			Yes	>12h	Yes	NA
IA-473	20/6/2024	65	European	2.9	Female	2	No			Yes	>12h	Yes	NA
IA-474	20/6/2024	65	European	3	Male (neutered)	6	No			Yes	>12h	Yes	NA
IA-475	19/6/2024	65	European	3.2	Female	3	No			Yes	>12h	Yes	NA
IA-476	17/6/2024	65	European	2.94	Female (neutered)	4	No			Yes	>12h	No	
IA-477	17/6/2024	65	European	3.8	Male	2	No			Yes	>12h	No	
IA-478	5/6/2024	65	European	5.5	Male (neutered)	7.5	No			Yes		No	
IA-479	5/6/2024	65	European	3	Female	1	No			Yes	>12h	Yes	NA
IA-480	4/6/2024	65	European	6.5	Male (neutered)	3	No			Yes	>12h	Yes	NA
IA-481	3/6/2024	65	European	3.4	Female	1.5	No			Yes	>12h	Yes	NA
IA-482	26/08/24	46	Russian blue	2.7	Female (neutered)	12	No			No	1-12h	No	Occasional
IA-483	26/08/24	46	European		Female (neutered)	10	No			No	>12h	No	Frequent
IA-484	24/8/2024	46	European	3.5	Female (neutered)	14	No			No	1-12h	No	Frequent
IA-485	24/8/2024	46	European	2.5	Female (neutered)	1	No			No	>12h	No	Frequent
IA-486	28/8/2024	46	European	3.7	Male (neutered)	12	No			No	1-12h	No	Frequent

IA-487	29/8/2024	46	Persian (cross	4.6	Male	11	No			No	>12h	No	Frequent
IA-488	30/8/2024	46	European		Female (neutered)	10	No			No	1-12h	No	Occasional
IA-489	29/8/2024	46	European	6	Male (neutered)	14	No			No	>12h	No	Frequent
IA-490	30/8/2024	46	European	5	Male (neutered)	6	No			No	1-12h	No	Occasional
IA-491	2/9/2024	46	European		Female (neutered)	18	No			No	<1h	No	None
IA-492	9/9/2024	46	European	3.35	Female (neutered)	9	No			No	1-12h	No	Occasional
IA-493	3/3/2024	46	European	3	Female (neutered)	14	No			No	1-12h	No	Occasional
IA-494	4/9/2024	46	European		Female (neutered)	6	No			No	1-12h	No	Frequent
IA-495		46	European		Female (neutered)	12	No			No	1-12h	No	Occasional
IA-496	18/9/2024	46	European	4.8	Female (neutered)	8	No			No	1-12h	No	Occasional
IA-497	4/9/2024	46	European	3.4	Male (neutered)	8	No			No	1-12h	No	Occasional
IA-498	8/9/2024	46	European		Male (neutered)	2	No			No	1-12h	No	Frequent
IA-499	10/9/2024	46	European	4.4	Male (neutered)	14	No			No	>12h	No	Occasional
IA-500	19/9/2024	46	European	6.2	Male (neutered)	11	No			No	1-12h	No	Occasional
IA-501	21/9/2024	46	European	3.5	Female	3	No			No	>12h	Yes	NA
IA-502	20/2/2024	18	European	4	Male	4	No			Yes	>12h	No	Frequent
IA-503	14/2/2024	18	European	4.5	Female	5	No			Yes	1-12h	No	Frequent
IA-504	13/2/2024	18	European	4.7	Male (neutered)	16	No			Yes	1-12h	No	None
IA-505	16/2/2024	18	European	4.9	Female	3	No			Yes	1-12h	No	Frequent
IA-506	21/2/2024	18	European	4.5	Male	2	No			Yes	>12h	No	Occasional
IA-507	4/3/2024	18	European	5	Male	6	No			Yes	>12h	No	Occasional
IA-508	16/4/2024	18	European	3.5	Female (neutered)	14	Yes	>'1/1280	1/320	Yes	1-12h	No	Frequent
IA-509	20/4/2024	18	European	3	Female	7	No			Yes	>12h	No	Frequent
IA-510	20/4/2024	18	Main Coon	3.8	Male (neutered)	6	No			Yes	>12h	No	Occasional
IA-511	29/3/2024	18	European	4	Male (neutered)	14	No			Yes	1-12h	No	Occasional
IA-512	29/3/2024	18	European	3	Male (neutered)	12	No			Yes	1-12h	No	Occasional
IA-513	30/5/2024	18	European	7	Male (neutered)	12	No			Yes	1-12h	No	None
IA-514	28/6/2024	18	European	5	Female	2	No			Yes	>12h	No	Frequent
IA-515	28/6/2024	18	European	4	Male	8	No			Yes	>12h	No	Frequent
IA-516	29/6/2024	18	European	4.8	Male (neutered)	5	No			Yes	1-12h	No	Occasional
IA-517	1/7/2024	18	European	4.5	Female	3	No			Yes	>12h	No	Frequent
IA-518	3/7/2024	18	European	3.5	Female	4.5	No			Yes	>12h	No	Frequent
IA-519	3/7/2024	18	European	3	Female	3.5	Yes	1/640	1/320	Yes	1-12h	No	Occasional
IA-520	3/7/2024	18	European	4	Male (neutered)	5	No			Yes	>12h	No	Occasional
IA-521	26/7/2024	18	European	6.7	Male (neutered)	14	No			Yes	1-12h	No	None
IA-522	30/7/2024	18	European	4	Male (neutered)	12	No			Yes	1-12h	No	Occasional
IA-523	31/8/2024	18	European	5	Male (neutered)	10	No			Yes	>12h	No	Occasional
IA-524		18					No			Yes		No	
IA-525		18					No			Yes		No	
IA-526	12/9/2024	46	European	4	Male (neutered)	11	No			No	1-12h	No	Occasional
IA-527	12/9/2024	46	European	5.6	Male (neutered)	12	No			No	1-12h	No	Occasional
IA-528	16/9/2024	46	European		Male (neutered)	10	No			No	1-12h	No	Occasional
IA-529	16/9/2024	46	European	4.5	Male (neutered)	5	No			No	1-12h	No	Occasional
IA-530	28/9/2024	46	European	4.75	Male	2	No			No	>12h	No	Frequent
IA-531	05/10/24	46	European		Male (neutered)	15	No			No	1-12h	No	Occasional
IA-532	06/10/24	46	Siamese	6.4	Male (neutered)	7	No			No	1-12h	No	Occasional
IA-533	9/9/2024	16	European	5	Female	9	No			No	1-12h	No	Occasional
IA-534	11/9/2024	16	European	4	Male	2	No			No	1-12h	No	None
IA-535	14/9/2024	16	European	4	Male (neutered)	13	No			No	1-12h	No	Occasional
IA-536	16/9/2024	16	European	3.95	Male	3	No			No	1-12h	No	Occasional
IA-537	16/9/2024	16	European	3	Female		No			No	<1h	No	None
IA-538	20/9/2024	16	European	5.5	Female	8	No			No	<1h	No	None
IA-539	25/9/2024	16	European	5.05	Female		No			No	<1h	No	None
IA-540	25/9/2024	16	European	3.75	Female (neutered)	1.2	No			No	1-12h	No	None
IA-541	25/9/2024	16	European	5.05	Male	8	No			No	>12h	No	Frequent
IA-542	28/9/2024	16	European	4.45	Male	3	No			No	<1h	No	Occasional
IA-543	30/9/2024	16	European	3.75	Female	10	No			No	>12h	No	Occasional
IA-544	30/9/2024	16	European	4	Female	2	No			No	1-12h	No	Occasional
IA-545	26/09/24	44	European	6	Male (neutered)	4	No			Yes	>12h	No	Occasional
IA-546	27/09/24	44	European	4.4	Female (neutered)	7	No			Yes	>12h	No	None
IA-547	28/09/24	44	European	4.2	Male (neutered)	4.5	No			Yes	1-12h	No	Occasional

IA-548	27/09/24	44	European		Female (neutered)	5	No			Yes	1-12h	No	Occasional
IA-549	28/09/24	44	European	4.7	Male (neutered)	15	No			Yes	1-12h	No	Frequent
IA-550	18/09/24	44	European	5.5	Male (neutered)	13.8	No			Yes	>12h	No	None
IA-551	01/10/24	44	European	4.2	Female (neutered)	9	No			Yes	>12h	No	Occasional
IA-552	03/10/24	44	European	2.5	Male (neutered)	8	No			Yes	>12h	No	Occasional
IA-553	03/10/24	44	European	6	Male (neutered)	8.4	No			Yes	1-12h	No	Occasional
IA-554	10/10/24	44	European	5.6	Male (neutered)	7.4	No			Yes	1-12h	No	Occasional
IA-555	19/03/24	85	European	3.2	Female	2	No			Yes	>12h	No	Frequent
IA-556	19/04/24	85	European	3.75	Female (neutered)	8	No			Yes	>12h	No	Frequent
IA-557	20/04/24	85	European	4.8	Female (neutered)	3	No			Yes	>12h	No	Frequent
IA-558	14/06/24	85	European	2.6	Female	2	No			Yes	>12h	No	Occasional
IA-559	19/03/24	85	European	2	Female	0.5	No			Yes	>12h	No	Frequent
IA-560	28/05/24	85	European	3	Female	1	No			Yes	>12h	No	Occasional
IA-561	28/05/24	85	European	5	Male	7	No			Yes	>12h	No	Occasional
IA-562	28/05/24	85	European	3.2	Male	1	No			Yes	>12h	No	Frequent
IA-563	29/05/24	85	European	3	Male	2	Yes	1/20	1/20	Yes	>12h	Yes	NA
IA-564	13/06/24	85	European	3	Male (neutered)	2	No			Yes	>12h	No	Occasional
IA-565	13/06/24	85	European		Male (neutered)	0.8	No			Yes		No	
IA-566	30/09/24	85	European	3.7	Male	2	No			Yes	1-12h	No	Occasional
IA-567	30/09/24	85	European	3.1	Male (neutered)	3	No			Yes	1-12h	No	Occasional
IA-568	17/10/24	85	European	3.5	Male		No			Yes	>12h	No	Frequent
IA-569	15/10/24	35	European	3.6	Female (neutered)	12	No			Yes	<1h	No	Occasional
IA-570	16/10/24	35	European	4.4	Female	9	No			Yes	>12h	No	Frequent
IA-571	16/10/24	35	European	5.1	Male (neutered)	5	No			Yes	1-12h	No	None
IA-572	16/10/24	35	European	4.9	Female (neutered)	10	No			Yes	<1h	No	None
IA-573	16/10/24	35	European	3.8	Male (neutered)	5.9	No			Yes	1-12h	No	Occasional
IA-574	21/10/24	35	European	4.9	Male (neutered)	0.18	No			Yes	>12h	No	Occasional
IA-575	22/10/24	35	European	3.4	Male (neutered)	11	No			Yes	1-12h	No	Occasional
IA-576	23/10/24	35	European	3.5	Female (neutered)	16	No			Yes	<1h	No	None
IA-577	24/10/24	35	European	5	Male (neutered)	10	No			Yes	1-12h	No	Occasional
IA-578	24/10/24	35	European	3.7	Male (neutered)	16	No			Yes	<1h	No	Occasional
IA-579	31/05/24	3	European	3.5	Female (neutered)	5	No			No	>12h	No	Occasional
IA-580	04/06/24	3	European	5.12	Male (neutered)	7	No			No	1-12h	No	Occasional
IA-581	05/06/24	3	European	3.38	Female (neutered)	8	No			No	>12h	No	Frequent
IA-582	06/06/24	3	European	7.6	Male (neutered)	6	No			No	1-12h	No	Occasional
IA-583	19/06/24	3	European	6.8	Male (neutered)	14	No			No	1-12h	No	Occasional
IA-584	20/06/24	3	European	2.7	Male (neutered)	2	No			No	1-12h	No	Frequent
IA-585	25/06/24	3	European	3	Female (neutered)	1	No			No	1-12h	No	Occasional
IA-586	26/06/24	3	European	3.82	Female (neutered)	7	No			No	>12h	No	Frequent
IA-587	27/06/24	3		5.1	Female (neutered)	6	No			No	>12h	No	Occasional
IA-588	03/07/24	3	European	4	Male (neutered)	1	No			No	1-12h	No	Frequent
IA-589	03/07/24	3	European	5.5	Male (neutered)	7	No			No		No	Frequent
IA-590	04/07/24	3	European	2.9	Female (neutered)	4	No			No	1-12h	No	None
IA-591	06/07/24	3	European	7.4	Male (neutered)	8	No			No	1-12h	No	Frequent
IA-592	05/07/24	3		3.430	Female (neutered)	7	No			No	>12h	No	Occasional
IA-593	16/09/24	65	European	3.6	Male	2	No			Yes	>12h	Yes	NA
IA-594	18/09/24	65	European	4.5	Male	1.5	No			Yes	>12h	Yes	NA
IA-595	03/10/24	65	European	2.5	Female (neutered)	15	No			Yes	1-12h	No	
IA-596	04/10/24	65	European	5.2	Male		No			Yes		No	
IA-597	11/10/24	65	European	2.6	Female	2	No			Yes	1-12h	No	
IA-598	11/10/24	65	European	2.5	Female	1.5	No			Yes	>12h	Yes	NA
IA-599	11/10/24	65	European	2.8	Female	0.6	No			Yes	1-12h	No	
IA-600	11/10/24	65	European	2.8	Female	0.6	No			Yes	1-12h	No	
IA-601	11/10/24	65	European	3.7	Female	3	No			Yes	>12h	Yes	NA
IA-602	14/10/24	65	European		Male	17	No			Yes	1-12h	No	
IA-603	30/10/24	65	European	5	Male (neutered)	1	Yes	1/160	1/40	Yes	>12h	Yes	NA
IA-604	30/10/24	65	European	5.7	Male	5	No			Yes	>12h	Yes	NA
IA-605	13/11/24	65	European		Female (neutered)	6	No			Yes	>12h	Yes	NA
IA-606	27/11/24	65	European	3.5	Female	2	No			Yes	>12h	No	
IA-607	09/12/24	65	European	4	Male		No			Yes	1-12h	No	
IA-608	03/10/24	32	European	3	Female		No			Yes	>12h	No	

IA-609	15/07/24	32	European	5.35	Female (neutered)	10	No			Yes	>12h	No	Occasional
IA-610	09/07/24	32	European	5.2	Male (neutered)	8	No			Yes	1-12h	No	None
IA-611	27/06/24	32	European	4	Male	4	No			Yes	>12h	No	Frequent
IA-612	05/07/24	32	European	4.5	Female (neutered)	12	No			Yes	<1h	No	None
IA-613	09/07/24	32	European	3.5	Female (neutered)	11	No			Yes	1-12h	No	None
IA-614	09/09/24	32	European	4.8	Female (neutered)	15	No			Yes	1-12h	No	None
IA-615	07/10/24	32	European	5.2	Male (neutered)	8	No			Yes	>12h	No	Occasional
IA-616	07/10/24	32	European	6	Female (neutered)	3	No			Yes	1-12h	No	Occasional
IA-617	09/10/24	32	European	4.8	Female (neutered)	13	No			Yes	>12h	No	Frequent
IA-618	09/10/24	32	European		Male (neutered)		No			Yes	>12h	No	Occasional
IA-619	10/10/24	32	European	4	Female (neutered)	2	No			Yes	>12h	No	Occasional
IA-620	23/10/24	32	European	3.250	Female (neutered)	1	No			Yes	>12h	No	Occasional
IA-621	30/10/24	32	European	3.080	Female (neutered)	11	No			Yes	1-12h	No	Frequent
IA-622	31/10/24	35	European	2.7	Male (neutered)	0.5	No			Yes	1-12h	No	Occasional
IA-623	31/10/24	35	European	4.9	Male (neutered)	9	No			Yes	1-12h	No	Occasional
IA-624	15/10/24	35	European	1.7	Female (neutered)	0.6	No			Yes	1-12h	No	Occasional
IA-625	15/10/24	35	European	4.2	Male	1.5	No			Yes	1-12h	No	Frequent
IA-626	15/10/24	35	European	4.1	Male (neutered)	17	Yes	1/40	1/320	Yes	1-12h	No	Frequent
IA-627	08/10/24	35	European	2.15	Female	0.5	No			Yes	1-12h	No	Occasional
IA-628	07/10/24	35	European	3.1	Female (neutered)	7	No			Yes	1-12h	No	Occasional
IA-629	09/12/24	44	European	3.350	Male (neutered)		No			Yes	>12h	No	Occasional
IA-630	27/12/24	44	European		Male		No			Yes	>12h	Yes	NA
IA-631	20/11/24	44	European	5.1	Female	3	No			Yes	>12h	Yes	NA
IA-632	15/11/24	44	European		Female		No			Yes	>12h	No	Occasional
IA-633	05/11/24	44	European	4.65	Male (neutered)	6	No			Yes	1-12h	No	Occasional
IA-634	07/11/24	44	European	2.72	Female	1	No			Yes	>12h	No	Occasional
IA-635	07/11/24	44	European	3.1	Female	1	No			Yes	>12h	No	Occasional
IA-636	05/11/24	44	European	2.840	Female	2	No			Yes	>12h	Yes	NA
IA-637	25/10/24	44	European	3.15	Male	1.5	No			Yes	>12h	No	Frequent
IA-638	25/10/24	44	European	2.86	Female	1.5	No			Yes	>12h	No	Frequent
IA-639	22/10/24	44	European	4.8	Male (neutered)	6.5	No			Yes	1-12h	No	Occasional
IA-640	19/10/24	44	European	4.3	Male (neutered)	16	No			Yes	1-12h	No	Occasional
IA-641	19/10/24	44	European	5.3	Male (neutered)	6	Yes	1/320	0	Yes	1-12h	No	Occasional
IA-642	16/10/24	44	European	3.2	Female (neutered)	2	No			Yes	1-12h	No	Frequent
IA-643	18/10/24	44	European	8.5	Male (neutered)	9.5	No			Yes	1-12h	No	Occasional
IA-644	16/10/24	44	European	5.8	Male (neutered)	10	No			Yes	1-12h	No	Occasional
IA-645	16/10/24	44	European	3.8	Female (neutered)	3	No			Yes	>12h	No	Frequent
IA-646	18/10/24	44	European		Male		No			Yes	>12h	No	Frequent
IA-647	09/10/24	44	European	4.65	Female	14	No			Yes	<1h	No	Occasional
IA-648	20/09/24	44	European	4	Male	5	Yes	1/640	0	Yes	1-12h	No	Occasional
IA-649	23/09/24	44	European	4.01	Male (neutered)	2	No			Yes	1-12h	No	Occasional
IA-650	17/09/24	44	European	2.9	Female	3	No			Yes	>12h	No	Occasional
IA-651	02/10/24	44	European	3.6	Female (neutered)	1	No			Yes	>12h	No	None
IA-652	09/12/24	44	European	2.83	Female (neutered)		No			Yes	>12h	No	Occasional
IA-653	16/01/2025	49		4.5	Male (neutered)	2	No			Yes	<1h	No	
IA-654	16/01/2025	49	European		Female (neutered)	4	No			Yes	1-12h	No	
IA-655	13/01/2025	49	European	4.295	Male (neutered)	1.5	No			Yes		No	
IA-656	13/01/2025	49	European	5.225	Male (neutered)	5	No			Yes		No	
IA-657	13/01/2025	49	European	4.385	Male (neutered)	2.5	No			Yes		No	
IA-658	16/01/2025	49	European	2.6	Female (neutered)		No			Yes		No	
IA-659	16/01/2025	49	European	4.6	Female	5	No			Yes	1-12h	No	
IA-660	13/01/2025	49	European	6.4	Male (neutered)	6.5	No			Yes	1-12h	No	
IA-661	31/12/2024	49	European	5.95	Male	5.5	No			Yes	>12h	No	
IA-662	26/12/2024	49	European	4.35	Européen	9.5	No			Yes	1-12h	No	
IA-663	23/12/2024	49	European	4.7	Male (neutered)	14.5	No			Yes	1-12h	No	
IA-664	23/12/2024	49	European	2.37	Female (neutered)	0.6	No			Yes	1-12h	No	
IA-665		49	European	6	Male	11.5	No			Yes	1-12h	No	Occasional
IA-666	13/12/2024	49		3.55	Female (neutered)	1	No			Yes	1-12h	No	None
IA-667	17/12/2024	49	European	3.95	Européen	0.9	No			Yes	1-12h	No	Occasional
IA-668	17/12/2024	49		2.875	Female (neutered)	1	No			Yes	>12h	No	
IA-669	19/12/2024	49	European		Female (neutered)	4	No			Yes	1-12h	No	

IA-670	19/12/2024	49	European	4.5	Male (neutered)	2.5	No			Yes	1-12h	No	
IA-671	19/12/2024	49	European	2.8	Female (neutered)	0.7	No			Yes	1-12h	No	
IA-672	17/12/2024	49	European	4	Male	2	No			Yes	>12h	No	Frequent
IA-673	16/12/2024	49	European	4.7	Female (neutered)	16.5	No			Yes	<1h	No	None
IA-674	13/12/2024	49	European	5.1	Male (neutered)	6	No			Yes	1-12h	No	
IA-675	12/12/2024	49	European	5.7	Male (neutered)	13	No			Yes		No	
IA-676	26/12/2024	49	European	5.7	Female (neutered)	10.5	No			Yes	1-12h	No	
IA-677	07/11/2024	33	European	4.3	Male (neutered)	13	No			Yes	1-12h	No	Occasional
IA-678	03/09/2024	33	European	7.1	Female (neutered)	6	No			Yes	1-12h	No	Occasional
IA-679	12/12/2024	33	European	4.3	Male (neutered)	12	No			Yes	1-12h	No	None
IA-680	30/12/2024	33	European	3.4	Female (neutered)	1	No			Yes	1-12h	No	Occasional
IA-681	22/08/2024	33	European	5.8	Male (neutered)	2	No			Yes	>12h	No	None
IA-682	08/10/2024	33	European		Female (neutered)		No			Yes		No	
IA-683	08/11/2024	85	European	6.1	Male (neutered)	11	No			Yes	>12h	No	Occasional
IA-684	30/09/2024	85	European	4.5	Male (neutered)	5	No			Yes	>12h	No	Occasional
IA-685	04/10/2024	85	European	2.5	Female	5	No			Yes	>12h	No	Frequent
IA-686	16/10/2024	85	European	3.83	Male (neutered)	5	No			Yes	>12h	No	Frequent
IA-687	12/11/2024	85	European	4	Female (neutered)	12	No			Yes	>12h	No	Occasional
IA-688	11/10/2024	85	European				No			Yes	>12h	No	Frequent
IA-689	08/11/2024	85	European		Male (neutered)		No			Yes	>12h	No	Occasional
IA-690	13/11/2024	85					No			Yes	>12h	No	Occasional
IA-691	19/11/2024	85					No			Yes		No	
IA-692	22/11/2024	20	European	5.3	Male (neutered)	6.5	No			No	1-12h	No	None
IA-693	20/11/2024	20	European	4.2	Male (neutered)	14.5	No			No	1-12h	No	Occasional
IA-694	08/11/2024	20	European	4.4	Male (neutered)	2	No			No	1-12h	No	Occasional
IA-695	07/11/2024	20	Persan	2.8	Female (neutered)	12.5	No			No	<1h	No	None
IA-696	24/10/2024	20	European	6	Male (neutered)	7	No			No	1-12h	No	Occasional
IA-697	15/12/2024	20	European	3.5	Female (neutered)	15.5	No			No	<1h	No	None
IA-698	12/11/2024	20	European	5	Female (neutered)	15	No			No	1-12h	No	Occasional
IA-699	23/10/2024	20	European	4.1	Female (neutered)	17	No			No	1-12h	No	Entre 1 et 12h
IA-700	25/10/2024	20	European	7.2	Male (neutered)	10	No			No	<1h	No	None
IA-701	23/11/2024	20	European	4.8	Female (neutered)	13	No			No	1-12h	No	Occasional
IA-702	03/01/2025	20	European	7.5	Male (neutered)	10	No			No	1-12h	No	Occasional
IA-703	31/05/2024	20	European	4.82	Male	4	No			No	>12h	Yes	NA
IA-704	30/05/2024	20	European	4.75	Male (neutered)	6	No			No	>12h	Yes	NA
IA-705	13/05/2024	20	European	4.26	Male	8	No			No	>12h	Yes	NA
IA-706	18/04/2024	20	European	4.2	Male (neutered)	4	No			No	1-12h	No	Occasional
IA-707	16/04/2024	20	European	4.18	Male	10	No			No	>12h	Yes	NA
IA-708	26/03/2024	20	European	4.28	Male (neutered)	6.5	No			No	>12h	Yes	NA
IA-709	26/03/2024	20	European	2.9	Male	10	No			No	>12h	Yes	NA
IA-710	26/03/2024	20	European	4.35	Male (neutered)	4.5	No			No	>12h	Yes	NA
IA-711	26/02/2024	20	European	3.6	Male (neutered)	7	No			No	>12h	Yes	NA
IA-712	28/02/2024	20	European	4.1	Male	10	No			No	>12h	Yes	NA
IA-713	14/05/2024	20	European	2.94	Male (neutered)	2	No			No	>12h	Yes	NA
IA-714	14/05/2024	20	European	4.5	Male	10	No			No	>12h	Yes	NA
IA-715	28/10/2024	13	European	3.5	Male	1	No			Yes	>12h	Yes	NA
IA-716	28/10/2024	13	European	3.2	Female	1	No			Yes	>12h	Yes	NA
IA-717	28/10/2024	13	European	2.9	Female	0.6	No			Yes	1-12h	No	
IA-718	29/10/2024	13	European	4.9	Female (neutered)	10.5	No			Yes	<1h	No	None
IA-719	30/10/2024	13	European	2.9	Male (neutered)	10.5	No			Yes	1-12h	No	None
IA-720	30/10/2024	13	European	3.9	Unknown	9	No			Yes		No	
IA-721	04/11/2024	13	European	4.1	Female (neutered)	9.5	No			Yes	>12h	No	Occasional
IA-722	18/01/2025	13	European	5.5	Female		No			Yes	>12h	Yes	NA
IA-723	24/01/2025	13	European		Female		No			Yes	>12h	Yes	NA
IA-724	25/01/2025	13	European		Female		No			Yes	>12h	Yes	NA
IA-725	14/08/2024	17	European		Female (neutered)	3	No			Yes	>12h	No	Occasional
IA-726	14/08/2024	17	European	6	Male (neutered)	10	No			Yes	1-12h	No	None
IA-727	22/08/2024	17	European	2.5	Female (neutered)	8	No			Yes	1-12h	No	Occasional
IA-728	11/09/2024	17	European	3	Male (neutered)	0.5	No			Yes	1-12h	No	Occasional
